# Explainable machine learning on baseline MRI predicts multiple sclerosis trajectory descriptors

**DOI:** 10.1371/journal.pone.0306999

**Published:** 2024-07-16

**Authors:** Silvia Campanioni, César Veiga, José María Prieto-González, José A. González-Nóvoa, Laura Busto, Carlos Martinez, Miguel Alberte-Woodward, Jesús García de Soto, Jessica Pouso-Diz, María de los Ángeles Fernández Ceballos, Roberto Carlos Agis-Balboa

**Affiliations:** 1 Galicia Sur Health Research Institute (IIS Galicia Sur), Cardiovascular Research Group, Vigo, Spain; 2 Health Research Institute of Santiago de Compostela (IDIS), Translational Research in Neurological Diseases Group, Santiago University Hospital Complex, SERGAS-USC, Santiago de Compostela, Spain; 3 Neuro Epigenetics Lab, Health Research Institute of Santiago de Compostela (IDIS), Santiago University Hospital Complex, Santiago de Compostela, Spain; 4 Neurology Service, Santiago University Hospital Complex, Santiago de Compostela, Spain; University of Bahrain, BAHRAIN

## Abstract

Multiple sclerosis (MS) is a multifaceted neurological condition characterized by challenges in timely diagnosis and personalized patient management. The application of Artificial Intelligence (AI) to MS holds promises for early detection, accurate diagnosis, and predictive modeling. The objectives of this study are: 1) to propose new MS trajectory descriptors that could be employed in Machine Learning (ML) regressors and classifiers to predict patient evolution; 2) to explore the contribution of ML models in discerning MS trajectory descriptors using only baseline Magnetic Resonance Imaging (MRI) studies. This study involved 446 MS patients who had a baseline MRI, at least two measurements of Expanded Disability Status Scale (EDSS), and a 1-year follow-up. Patients were divided into two groups: 1) for model development and 2) for evaluation. Three descriptors: *β*_1_, *β*_2_, and EDSS(t), were related to baseline MRI parameters using regression and classification XGBoost models. Shapley Additive Explanations (SHAP) analysis enhanced model transparency by identifying influential features. The results of this study demonstrate the potential of AI in predicting MS progression using the proposed patient trajectories and baseline MRI scans, outperforming classic Multiple Linear Regression (MLR) methods. In conclusion, MS trajectory descriptors are crucial; incorporating AI analysis into MRI assessments presents promising opportunities to advance predictive capabilities. SHAP analysis enhances model interpretation, revealing feature importance for clinical decisions.

## 1. Introduction

Multiple Sclerosis (MS) is a complex, long-lasting condition affecting the brain and spinal cord, leading to symptoms such as vision disturbances, impaired limb movement cognitive impairment, etc [1]. The disease typically progresses from a Relapsing-Remitting (RR) phase to a Secondary-Progressive (SP) phase, resulting in worsening health and irreversible disability [2,3]. The introduction of over fifteen approved disease-modifying treatments offers the potential to delay the onset of the SP phase significantly. However, the benefits must be carefully balanced against substantial risks, especially with the most potent medications [4].

Magnetic Resonance Imaging (MRI) images offer insights into brain and spinal cord lesions associated with MS, facilitating accurate diagnosis and disease tracking [5,6]. Various techniques and scoring systems, such as the Barkhof [7] and Paty [8] scores, quantitatively evaluate lesions and disease burden in the central nervous system but typically applied at a specific point in time and do not consider the patient’s complete medical history. To obtain a comprehensive understanding of MS, it is crucial to consider broader factors, including genetics, microbiota, lifestyle, and geographical settings [9].

The absence of established prognostic markers and reliable risk scores currently complicates the accurate prediction of disease trajectories for individual patients, which is particularly challenging given the availability of treatments that can slow disease progression but come with potential adverse effects [10,11]. Early prognosis of disease trajectories could enable personalized treatment strategies, particularly for higher-risk patients, and Artificial Intelligence (AI) algorithms are gaining momentum in addressing this need in neurology [12–17], to heighten diagnostic precision and refine patient care efficacy. Some studies have used Machine Learning (ML) to explore MS, for both diagnosis and prognosis, offering promising insights [14,16]. While many studies focus on cross-sectional perspective and identifying static patterns in the data, comprehending how MS changes over time requires a longitudinal perspective. This approach helps connect information across different time points and understand the holistic trajectory of each patient [17].

This paper presents a novel methodology for exploring the correlation between features extracted from baseline MRI and the trajectory of MS in terms of each patient’s Expanded Disability Status Scale (EDSS) [18]. To achieve this, three different models are proposed to describe these trajectories, and ML tools such as XGBoost [19] and, subsequently, Shapley Additive Explanations (SHAP) [20], are applied to improve the understanding of these relationships.

## 2. Materials

### 2.1 Dataset

**Fig 1:**
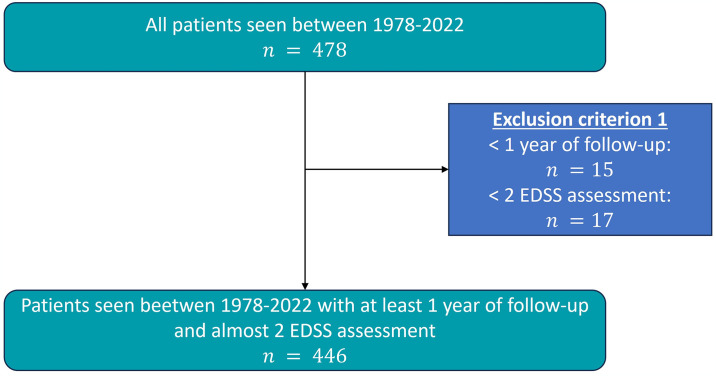
Illustration depicting the inclusion criteria used in the selection of participants for the studies.

In this study, a dataset comprising 478 patients from Galicia (North-Western Spain), was utilized. The exclusion criterion was based on patient follow-up, excluding those with less than 1 year of follow-up and those with fewer than two EDSS evaluations. After applying this selection criteria, a total of 446 records were employed for the analysis, with data collected from 1987 to 2022. Finally, it was ensured that each patient included in the analysis had an associated MRI. Fig 1 shows the cohort selection schema, and S1 Table shows the patient characteristics for the selected dataset. The data utilized, which was accessed for the first time on June 20, 2016, was approved by the Autonomous Committee of Research Ethics of Galicia under the code 2016/307. Informed consent was obtained from all participants prior to any data collection procedures.

**Fig 2:**
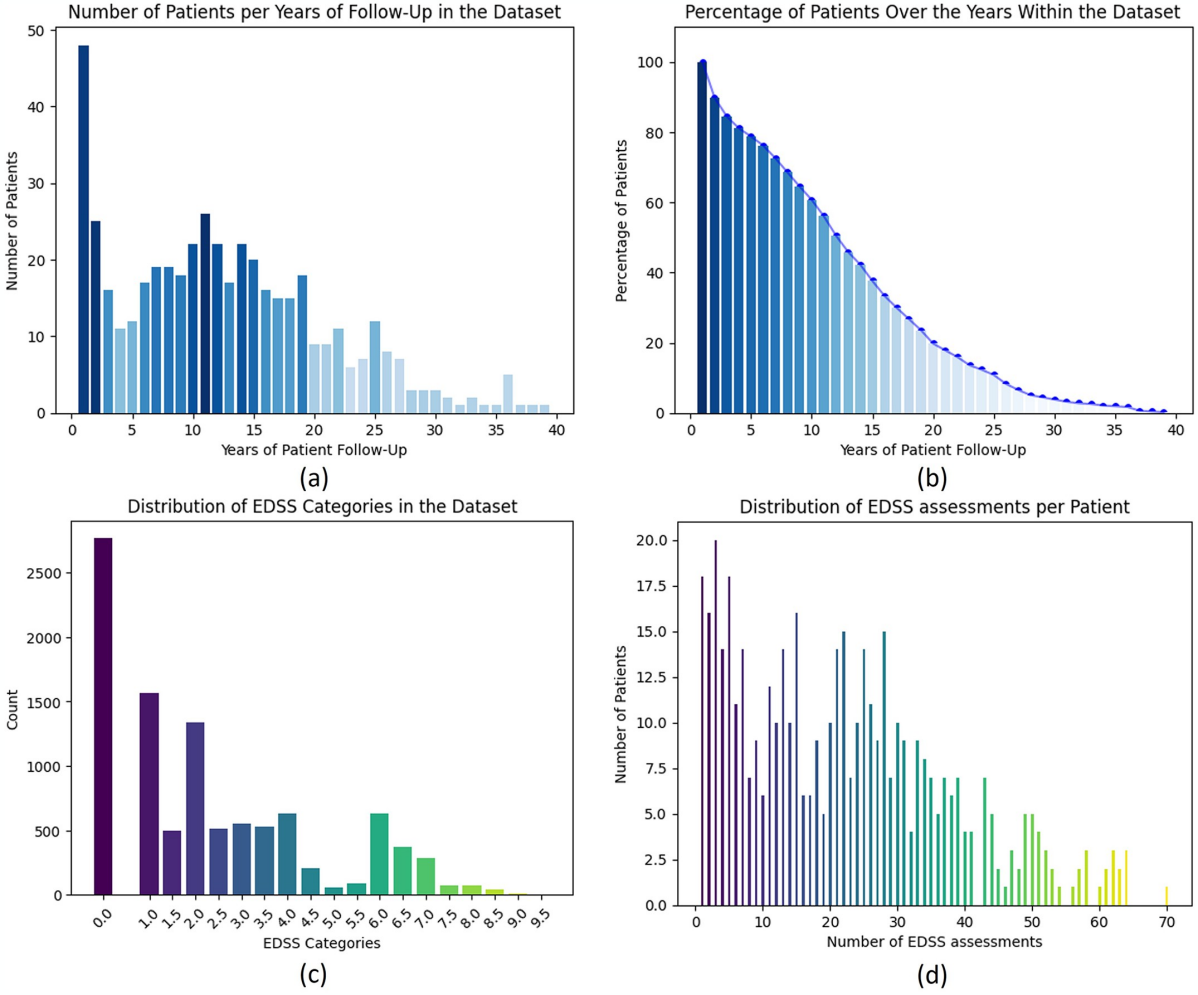
Temporal distribution of study cohort and follow-up duration. (A) Distribution of Patients Over Time: Follow-Up Duration in Days. (B) Temporal Distribution of Study Cohort: Percentage of Patients Across Follow-Up Duration in Years. (C) Distribution of the number of values of each EDSS category in the studied cohort. (D) Distribution of the number of determinations per patient in the studied cohort.

To examine our database in detail, it is essential to understand the temporal structure of the data. Fig 2A illustrates how the 446 patients included in the study are distributed categorized by the duration of their follow-up, measured in years. This representation helps us comprehend how patients are distributed concerning the duration of their follow-up in the database. On the other hand, in Fig 2B, we provide an alternative way to represent the percentage of our study cohort based on the duration of follow-up in years, offering insights into the length and quality of the collected data. Additionally, it is crucial to account for the distribution of EDSS scores in these MS patients. EDSS is a common clinical scale used to evaluate disability in MS patients, offering valuable insights into their clinical condition. Scores range from 0 (no disability) to 10 (severe disability). To illustrate the behaviour of EDSS within the dataset, Fig 2C displays the distribution of values in each EDSS category, while Fig 2D illustrates the distribution of EDSS determinations per patient. This provides a clearer understanding of the distribution of EDSS scores and the frequency of assessments in the dataset.

### 2.2 AI tools (boosting and explainability)

#### 2.2.1. XGBoost

Gradient Boosting is a powerful ensemble learning technique widely employed in ML for enhancing predictive models. This method sequentially builds a strong predictive model by combining the outputs of weak learners, usually decision trees. The algorithm minimizes an objective function, represented by Eq 1.

$$obj\left(\theta \right)=L\left(\theta \right)+\Omega (\theta )$$ (1)

where *L*(*θ*) is the training loss function measuring the model’s performance on the training data and *Ω*(*θ*) is the regularization term accounting for the complexity of the model.

XGBoost represents a highly efficient and scalable implementation of gradient boosted decision trees, systematically constructing additive models in a stepwise manner. This process leads to an ensemble of base learners exhibit superior prediction capabilities compared to individual classifiers. Each weak classifier is assigned a weight based on its prediction accuracy, allowing them to contribute effectively to the final prediction [19,20]. XGBoost, being an advanced implementation, introduces additional regularization measures to control overfitting. The objective function of XGBoost, aimed to be minimized, is given by Eq 2, and the regularization term is described in Eq 3.


$$obj\left(f_{t}\right)=\sum_{i=1}^{N}l\left(y_{i},\;{{\hat{y}}_{i}}^{t-1}+\;f_{t}(x_{i})\right)+\;\Omega \left(f_{t}\right)$$ (2)

$$\Omega \left(f_{t}\right)=\sum_{k=1}^{K}\gamma T+\;\frac{1}{2}\alpha \;\sum_{m=1}^{T}w_{m}^{2}$$ (3)

Where, *y*_*i*_ is the target value of the *i*-th instance, *ŷ*_*i*_ is predicted value at the *t*-th iteration, *f*_*t*_ (*x*_*i*_) is the additive decision tree model greedily added to improve performance, and *Ω*(*f*_*t*_) is a regularization term penalizing model complexity. *N* is the set of all samples in leaf m, *T* consists of the number of leaf nodes, *α* and *γ* are parameters of the tree. The score of leaf m is measured by *ω*_*m*_. This regularization procedure aims to compress the weights for many features to zero, facilitating feature selection.

#### 2.2.2 SHAP

Model interpretability poses a significant challenge in the realm of ML algorithms. To address this challenge, SHAP is recognized as a potent and commonly used tool in the realm of explainable AI, serving a pivotal function in elucidating the importance and influence of input features on model predictions [21,22]. The SHAP methodology is based on a unified framework rooted in cooperative game theory, assigning the contribution of each feature to the model’s output. Through a quantitative approach to assess the marginal impact of features, SHAP considers all feature combinations [23]. This facilitates a thorough comprehension of feature interactions and their combined impacts on predictions [24]. The mathematical expression representing SHAP values is given by Eq 4. This holistic perspective provides valuable insights into the inner workings of complex ML models, contributing to transparency and informed decision-making regarding model behavior and feature importance.

$$\emptyset_{i}=\frac{1}{\left|N\right|!}\;\sum_{S\subseteq N\{i\}}\frac{\left|S\right|!(\left|N\right|-\left|S\right|-1)}{N}\;\left[f\left(S\;⋃\;\left\{i\right\}\;\right)-f(S)\right]$$ (4)

where *f*(*S*) refers to the output of the XGBoost model, which is determined by a specific set of features denoted as S. The complete set of all features is represented by *N*. The final contributions, denoted as ∅_*i*_, are computed by averaging the contributions across all permutations of a feature set. Subsequently, the features are sequentially incorporated into the set, and their impact is reflected in the model’s output change.

## 3. Methods

**Fig 3:**
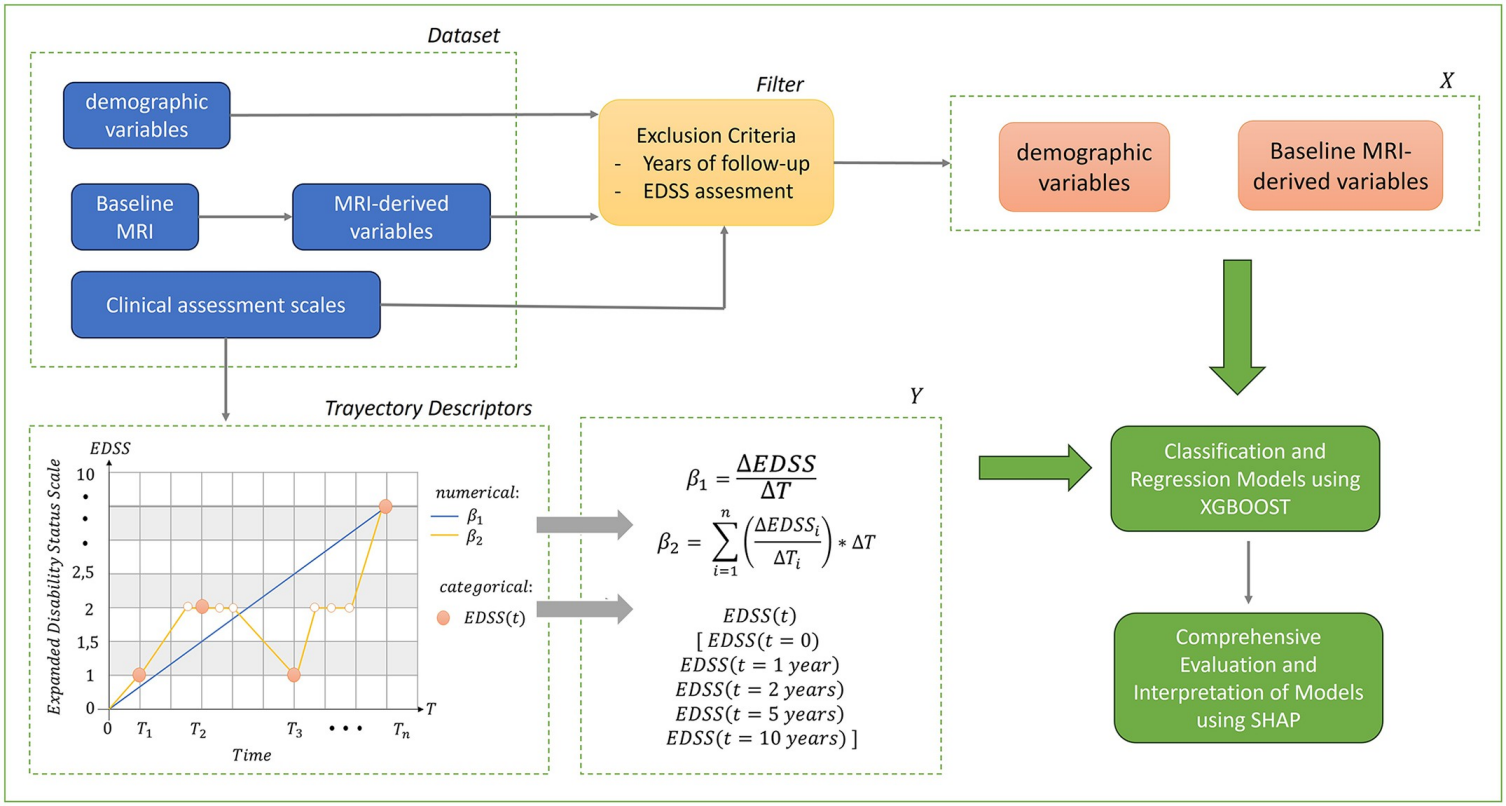
General schema of the methodology proposed in this work.

To assess the correlation between the features derived from the baseline MRI and the clinical trajectory of each patient (as assessed by EDSS), a new methodology has been developed, which is divided into several stages. The first stage involves cohort selection. The second stage is dedicated to proposing trajectory descriptors based on EDSS assessment. As a result, three descriptors were obtained (detailed in section 3.1), represented as *β*_1_, *β*_2_, and *EDSS*(*t*). Subsequently, we proceed to build AI models using these baseline MRI-derived features, gender, and age of MS onset to predict patient progression. Two models are considered for this purpose: the classical Linear Regressor (LR) [25] and the state-of-the-art XGBoost-based predictor (section 2.2.1). This allows for a dual evaluation: first, to determine if the ML approach (specifically XGBoost) outperforms the classical method (LR); and second, to validate the patient trajectory classification presented in the first section as patient descriptors. The final stage of the method is devoted to understanding, using explainable AI (referred to as SHAP in section 2.2.2), which are the main features of the model that predicts the trajectories. Fig 3 shows the methodology pipeline including all these stages.

### 3.1 Building the MS trajectory descriptors based on time dependent EDSS assessment variations

The first proposed trajectory descriptor aims to transform the patient’s categorical EDSS assessments into a single numerical variable representing the patient’s condition changes over time. This is achieved by combining measurements taken at various time points and normalizing them by the time interval (in days) between measurements. Mathematically, the first trajectory descriptor is based on the initial and final EDSS scores and is represented by Eq 5.


$$\beta_{1}=\frac{\Delta EDSS}{\;\Delta T\;}$$ (5)

Where ΔEDSS represents the variation in terms of EDSS between the initial and final measurements weighted by 0.5 for each category variation, whether increasing or decreasing, and the term ΔT corresponds to the difference (in days) between such measurements. The result, *β*_1_, corresponds to the *“slope”* of the line that connecting those points. This approach reduces the trajectory complexity of each patient to just two points (the beginning and the end).

Following the previous logic, a more comprehensive description of this score can be obtained by considering each of the EDSS assessments made for the patient. Using the same convention as in Eq 1 for Δ*T*, Eq 6 describes the second trajectory descriptor.


$$\beta_{2}=\sum_{i=1}^{n}\left(\frac{\Delta {EDSS}_{i}}{{\Delta T}_{i}}\right)*\Delta T=\sum_{i=1}^{n}\left(\frac{{EDSS\;}_{i}–{EDSS}_{i-1}\;}{\left(\;T_{i}-\;T_{i-1}\;\right)}\right)*\;{(T}_{n}-\;T_{i-1})$$ (6)

The term Δ*EDSS*_*i*_ means the variability in EDSS between two measurements, and the term Δ*T*_*i*_ corresponds to the number of days between two consecutive determinations. The summation covers EDSS variations from *i* = 1 to *n*, where *n* represents the last recorded change. Δ*EDSS*_*i*_ weighted by 0.5 on each category variation, increasing or decreasing. Consequently, the descriptor measures the time required to induce a change in EDSS values for a patient, treating them as a numerical variable.

The third trajectory descriptor aims to treat EDSS assessments directly as categorical. To consider the moment in time when these assessments are taken using this approach, a map of values at specific time points is created, which is the same for all patients. This is described in Eq 7.


EDSS(t)=[EDSS(t=0),EDSS(t=1),EDSS(t=2),EDSS(t=5),EDSS(t=10)] (7)

**Fig 4:**
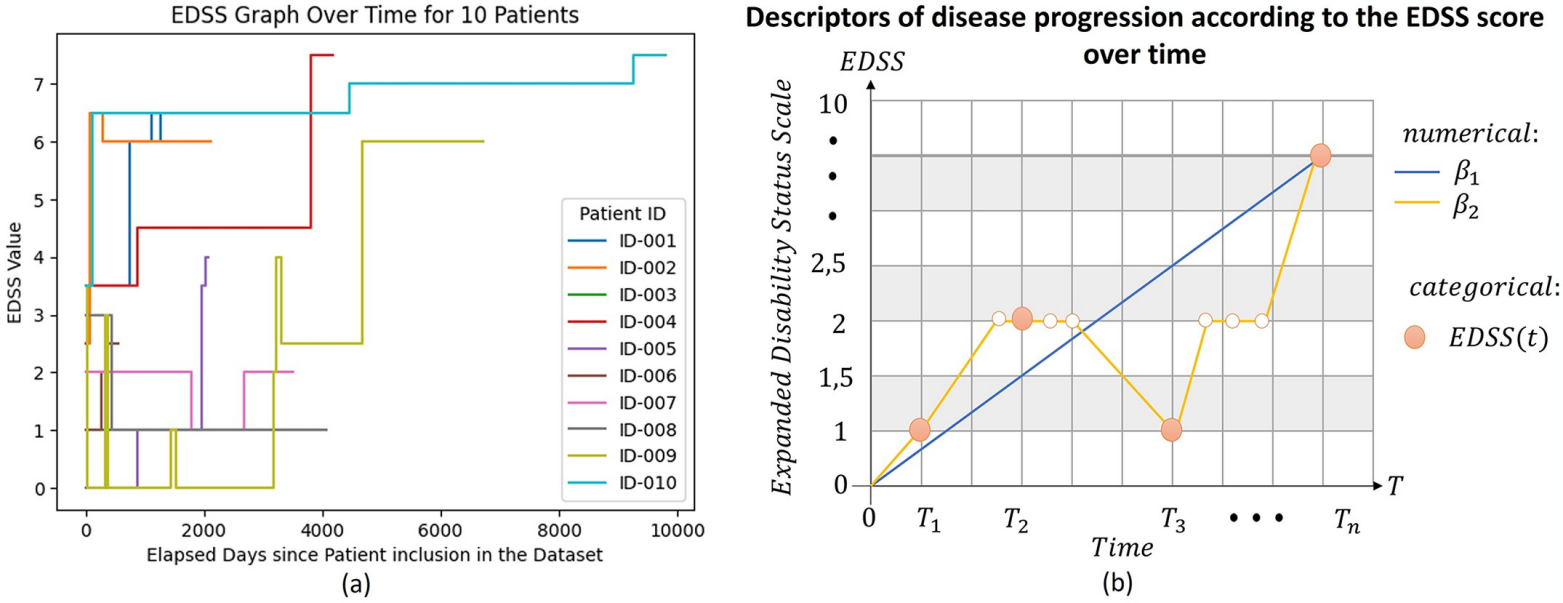
Description of the behaviour of the EDSS Variable in the dataset. (A) Evolution of EDSS for the first 10 patients of the study, where abscissa axis is time in days and ordinate, EDSS values. (B) Example of behaviour of possible descriptors of disease progression according to the EDSS score over time.

To describe patient trajectories using this model, we collect EDSS values at specific time intervals, including the initial assessment at t = 0, and subsequent evaluations at 1 year, 2 years, 5 years, and 10 years. This method enables us to predict the trajectory using classifiers that estimate the EDSS values at these specific time points. If a patient’s EDSS value matches that of the previous and subsequent assessments within a three-month interval in their medical history, we assume that it remains unchanged during that period. This assumption is particularly useful in cases where data for these time points are missing, as it provides additional values for the model to predict. Moreover, from a clinical point of view, the occurrence of a non-documented transient change (more often an increase than a decrease) of EDSS between two equal assessments is indeed a possibility in the clinical setting and would qualify as a (subclinical) relapse. Nevertheless, the purpose of this paper is to use baseline MRI features to predict the disability score (i.e., EDSS) at specific time points, not to foresee the annualized relapse rate (ARR). While the behaviour of ARR is a primary of most clinical trials in relapsing MS in the short and medium term (typically 1–2 years), disability is the most relevant feature in the long run (2–10 years) for either relapsing or progressive MS. These proposed descriptors aim to describe the progression of MS over time based on EDSS measures. Fig 4A depicts the variability of EDSS measures for a subset of the initial 10 patients in the study. Fig 4B showcases the behaviour of the disease progression descriptors concerning the EDSS scores over time.

### 3.2 AI (regressor and classifier) to predict MS trajectories

Once the data has been curated and pre-processed to enable integration into the AI workflow, two different sets of models are employed. The first one consists of the LR and the XGBoost regressors, used to predict the *β*_1_ and *β*_2_ trajectories, which represent the time required to change EDSS values. The second model utilizes Multiclass Logistic Regression (MLR) and the XGBoost classifiers to predict *EDSS*(*t*), forecasting the EDSS value at a specific moment (Eq 7), functioning as a classifier.

In the context of hyperparameter optimization, a crucial process for identifying and selecting parameter configurations that produce the best prediction results, the Bayesian approach was employed. Specifically, the Hyperopt Library [21] was utilized to optimize XGBoost (Eqs 2 and 3) leveraging Bayesian optimization.

For the analysis, the dataset is initially divided into two parts: one for model fitting and the other for evaluating predictor quality, using an 80/20 ratio. All models are fitted and hyperoptimized using the same set. Additionally, a five-fold cross-validation process is conducted, involving random resampling of the initial dataset split to assess the generalizability of the results. To address class distribution imbalance, stratified cross-validation is employed, ensuring that each fold maintains a representative proportion of the classes present. To evaluate whether the models are overfitting or underfitting, we compare the metrics (AUC-ROC, Sensitivity, Accuracy, Precision) between both the training and testing datasets. The values obtained permit us to conclude that neither overfitting nor underfitting occurs in the process.

## 4. Results

This section presents the results of three main experiments that explore the relationship between the progression of MS and the baseline MRI of each patient, using the dataset described in section 2.1. In the first experiment, trajectory descriptors were obtained using the different proposed models (section 3.1). The second experiment employed data from the baseline MRI to predict the patient trajectory based on the models obtained in the first experiment. In the third experiment, SHAP was utilized to analyse and understand the key features influencing the predictions of each model. The proposed method and analysis were implemented in Python using multiple libraries, including Scikit-learn, Matplotlib, NumPy, Pandas, Hyperopt, XGBoost, and SHAP.

### 4.1 Obtaining the trajectories descriptors

**Fig 5:**
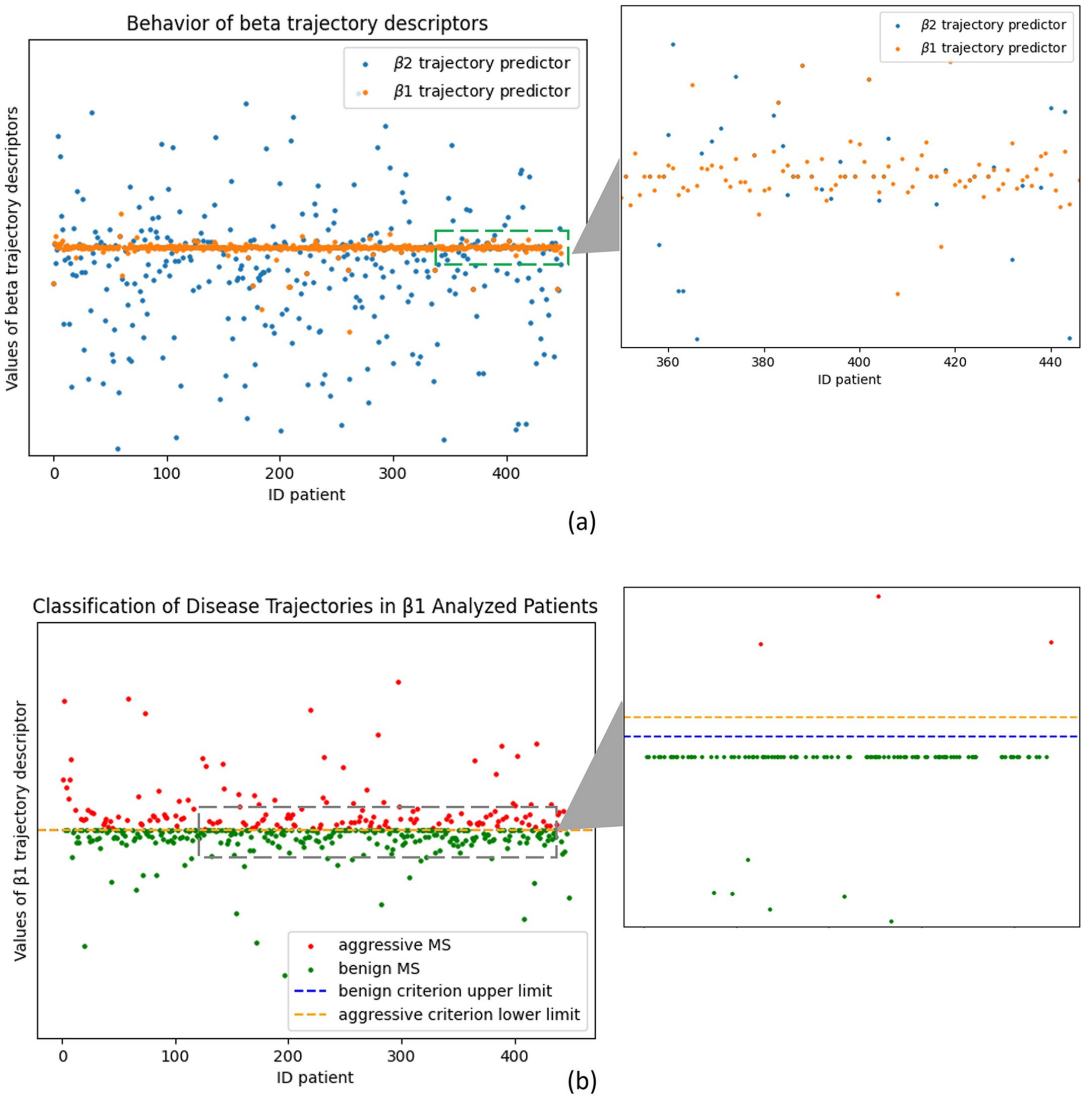
Behaviour of descriptors *β*_1_ and *β*_2_ in the dataset. (A) Results of *β*_1_ and f *β*_2_ for the Entire Patient Cohort. (B) Classification of disease trajectories in β1 analysed patients. In both at right a zoom of the area of interest.

The first experiment focuses on obtaining the descriptors for the dataset’s patients. *β*_1_ values, computed using Eq 5, show an average of 0.02 and a standard deviation of 1.23. For *β*_2_, values are extracted using Eq 6, revealing an average of -1.47, and a standard deviation of 12.76. Fig 5A displays the behaviour of *β*_1_ and *β*_2_ across all patients in our dataset. There is consensus in considering MS has followed a mild (termed ‘benign’ by some authors) course when EDSS score is ≤3.0, after a disease duration of at least 10 years [26], whereas aggressive MS might be defined as reaching an EDSS score of ⩾6.0 within 10 years of disease onset [27]. By calculating the β1 values for patients who meet these criteria, we’ve segmented the dataset into three groups: mild, average, and aggressive trajectories of the disease, as show in Fig 5B.

Regarding the extraction and quantification of *EDSS*(*t*) assessment for the specific moments as described in Eq 3. Results of this process are provided in Table 1, in terms of the number of patients in the study that has an EDSS value at each specific time.

**Table 1:** Distribution of patients that has each EDSS(t) for certain time values.

Parameter	EDSS Category											
	0	1	1.5	2	2.5	3	3.5	4	4.5	5	5.5	≥6
***EDSS*(0)**	47	52	15	125	39	60	58	18	3	0	3	26
***EDSS*(1)**	103	74	14	72	21	23	31	23	2	1	1	35
***EDSS*(2)**	96	63	19	60	22	24	25	26	5	1	1	36
***EDSS*(5)**	86	51	20	43	22	21	24	23	10	2	3	47
***EDSS*(10)**	68	37	9	25	14	11	17	19	6	3	5	65

*EDSS*(0):EDSS(t) descriptor assessment at time t = 0; *EDSS*(1): EDSS(t) descriptor assessment at time t = 1 year; *EDSS*(2): EDSS(t) descriptor assessment at time t = 2 years; *EDSS*(5): EDSS(t) descriptor assessment at time t = 5 years; *EDSS*(10): EDSS(t) descriptor assessment at time t = 10 years.

### 4.2. Predicting patient trajectory descriptors using baseline MRI

The dataset described in 2.1 was employed to evaluate the presented AI methods to predict the patient trajectories, as described in section 3.2.

#### 4.2.1 Regressor model

To predict the trajectory descriptors *β*_1_ and *β*_2_ based on the baseline MRI, patient age, and sex, two regression models were employed. In Table 2, a comparison is presented between the classic LR model and the XGBoost model. This comparison includes default hyperparameters and the best-performing Bayesian hyperparameter-tuned model, and it measures performance in terms of Mean Absolute Error (MAE). The results displayed on the table, demonstrate the potential for AI methods to significantly reduce prediction errors for both trajectory descriptors when compared to the classical LR method.

**Table 2:** Results of the Mean Absolute Error (MAE) obtained using three different regressor to predict the beta values of the dataset described in 2.2, using a split ratio of 80/20.

Predictor	MAE	
	*β*_1_	*β*_2_
Linear Regressor	0.25	7.22
XGBoost	0.19	6.87
XGBoost hyperoptimized	0.11	5.18

*Prediction of benignant and aggressive evolutions*. Following the prediction of the *β*_1_ trajectory descriptor, the results could be categorized according to the criteria described in section 4.1 to predict whether the disease course is classified as benign or aggressive. This method allows for the evaluation of the regressor’s ability to differentiate between the clinical categories of disease progression. Table 3 presents the results comparing the criteria forecasted by the regressor with the actual values from patients followed for ten years or more, belonging to the test group.

**Table 3:** Evaluation of disease progression prediction using various metrics, including AUC-ROC, sensitivity, precision, and accuracy.

Predictor	AUC-ROC	Sensitivity	Accuracy	Precision
**XGBoost hyperoptimized**	0.86	0.84	0.88	0.81

#### 4.2.2 Classifier model

**Fig 6:**
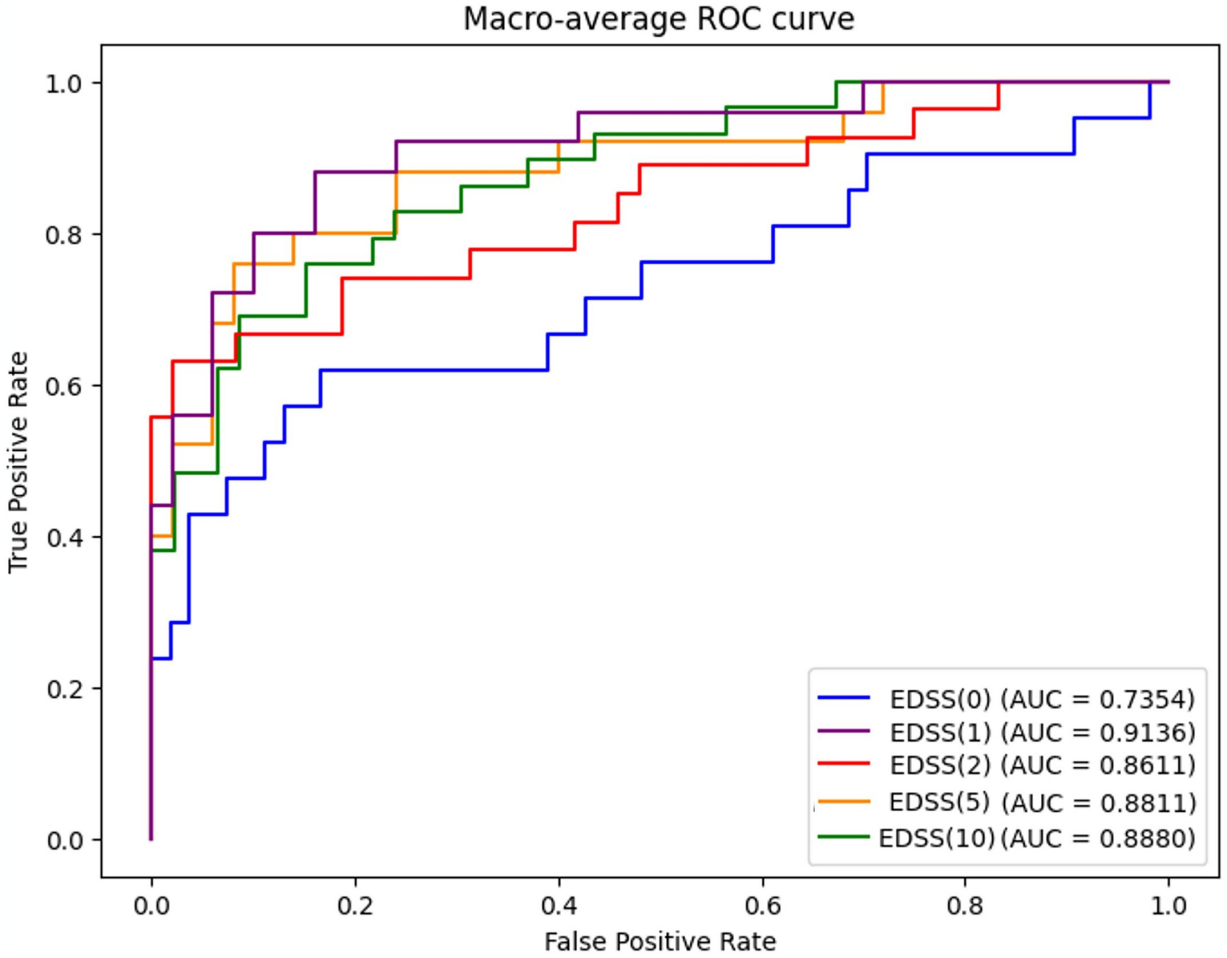
ROC Curves for XGBoost applied to each analysed *EDSS*(*t*) Timestamp. Table 4 presents the metrics derived from the testing dataset. To assess the model’s generalization performance, analogous metrics were calculated for the training dataset. These results are provided as supplementary material in S2 Table. A comparison between the metrics presented in Table 4 and those in S2 Table reveals a notable similarity in values for both the training and testing datasets. This congruence suggests that the models neither suffer from overfitting nor underfitting.

To make predictions based on the time descriptor *EDSS*(*t*), a classifier is needed. Table 4 compares the MLR with optimized XGBoost models using various metrics, including Area Under the Curve Receiver Operating Characteristic (AUC-ROC), Sensitivity, Precision, and Accuracy. The most promising results were achieved with the XGBoost model, with AUC-ROC values ranging from 0.7354 for *EDSS*(0) to the highest result of 0.9136 obtained for *EDSS*(1). Fig 6 displays the AUC-ROC curves generated by applying XGBoost to each *EDSS*(*t*) timestamp.

**Table 4:** Performance comparison of classifier models for predicting *EDSS*(*t*) on testing dataset.

*EDSS*(*t*)	CLS	AUC-ROC	Sensitivity	Accuracy	Precision
***EDSS*(0)**	**MLR** **XGBoost**	0.67 **0.74**	0.19 0.78	0.29 0.42	0.20 0.50
***EDSS*(1)**	**MLR** **XGBoost**	0.58 **0.91**	0.17 0.85	0.21 0.78	0.19 0.79
***EDSS*(2)**	**MLR** **XGBoost**	0.75 **0.86**	0.30 0.89	0.44 0.75	0.31 0.78
***EDSS*(5)**	**MLR** **XGBoost**	0.75 **0.88**	0.24 0.75	0.41 0.79	0.19 0.81
***EDSS*(10)**	**MLR** **XGBoost**	0.71 **0.89**	0.24 0.88	0.45 0.73	0.23 0.73

EDSS(0), EDSS(t) descriptor assessment at time t = 0; EDSS(1), EDSS(t) descriptor assessment at time t = 1 year; EDSS(2), EDSS(t) descriptor assessment at time t = 2 years; EDSS(5), EDSS(t) descriptor assessment at time t = 5 years; EDSS(10), EDSS(t) descriptor assessment at time t = 10 years; CLS, classifiers; AUC-ROC, Area Under the Receiver Operating Characteristic curve; MLR, Multiclass Logistic Regression.

### 4.3. Using SHAP to explain the ML models (regressor and classifier)

**Fig 7:**
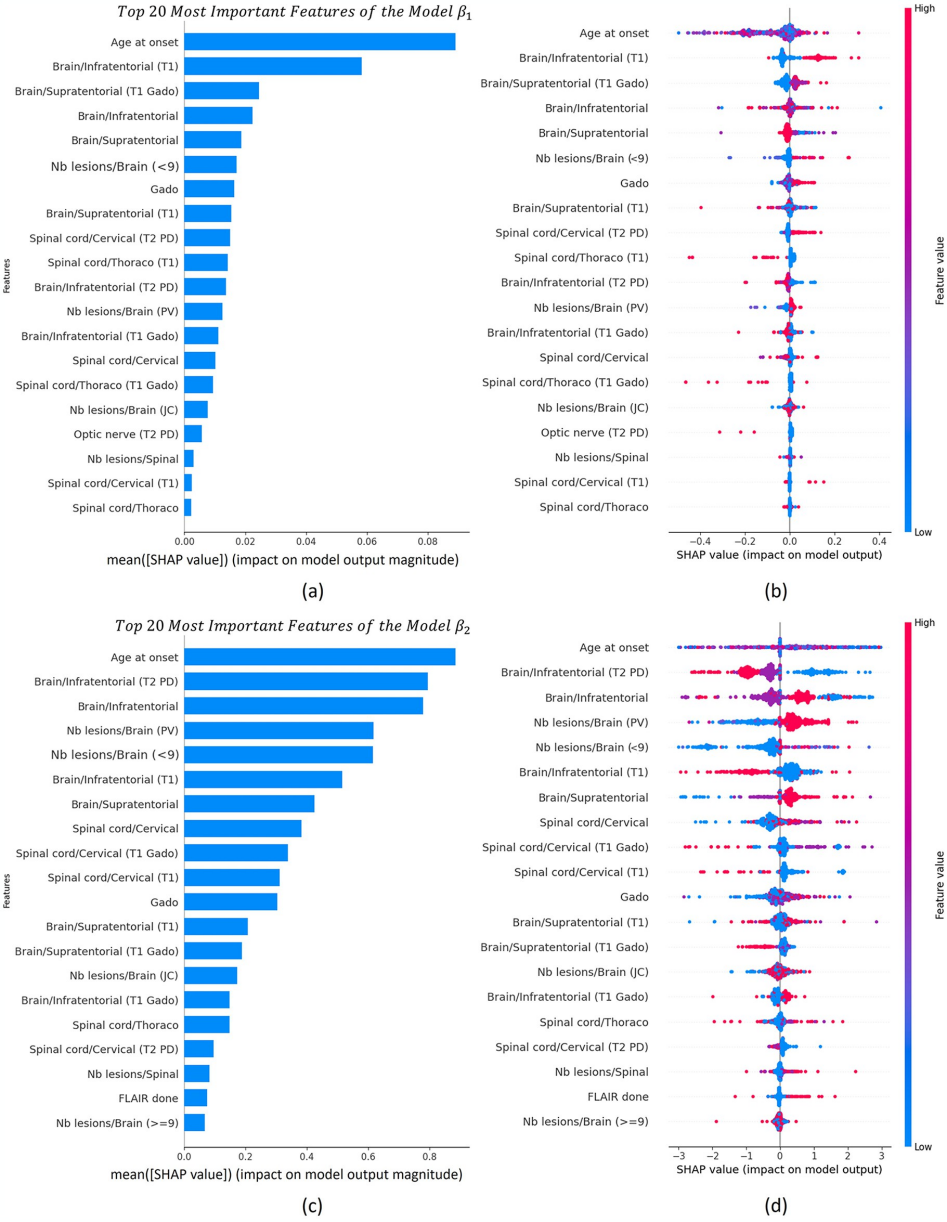
Relevance and SHAP analysis of the 20 most important features of the model XGBoost regressor to predict *β*_1_ y *β*_2_. (A-B) Relevance and SHAP analysis of the 20 most important clinical variables extracted from the XGBoost regressor to predict *β*_1_. (C-D) Relevance and SHAP analysis of the 20 most important clinical variables extracted from the XGBoost regressor to predict *β*_2_.

After obtaining the model, we employed SHAP (as described in section 2.2.2) to interpret the best-performing regressor identified in Table 2. In this case, we utilized the hyperoptimized version of XGBoost with predictors *β*_1_ and *β*_2_. Using this technique enables us to identify and rank the importance of features. The analysis reveals that "Age at onset" is the most crucial feature for predictors *β*_1_ and *β*_2_. Fig 7A and 7C displays plots of the ranking of the 20 most important variables. Fig 7B and 7D illustrates the impact of features on the model output for individuals in the validation dataset. The X-axis displays features sorted by the sum of SHAP value magnitudes across all samples, indicating higher importance at the extremes. The Y-axis shows how much each feature affects the model’s predictions using SHAP values. The colours, from red to blue, stand for high to low values of these features.

**Fig 8:**
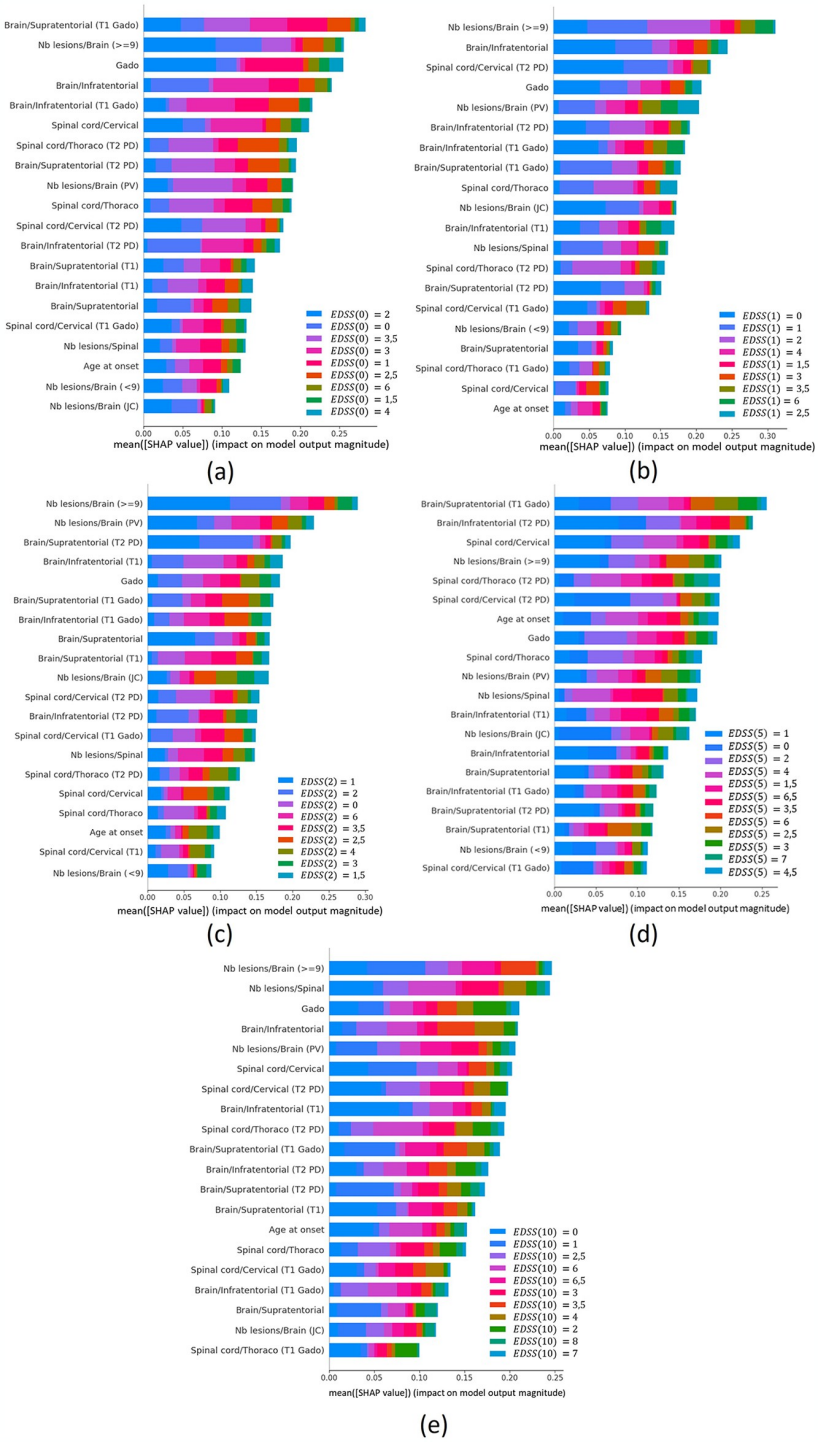
Relevance and SHAP analysis for those 20 most important clinical variables extracted from the XGBoost classifier to predict *EDSS*(*t*). (A) Analysis to predict *EDSS*(0). (B) Analysis to predict *EDSS*(1). (C) Analysis to predict *EDSS*(2). (D) Analysis to predict *EDSS*(5). (E) Analysis to predict *EDSS*(10).

In the section dedicated to the classifier, a similar approach to the regressor was followed, employing the SHAP technique to assess and elucidate the performance of ML models. Fig 8 displays the ranking of the top 20 features that exert the most influence on the model’s classification decisions, thereby contributing to a deeper understanding of variable importance and overall model performance.

In the SHAP analysis of the classifier, we observed variations in the influence of each variable across different classes and time points (as shown in Fig 8). Interestingly, while the age of onset exhibited reduced influence compared to other predictors, the number of lesions greater than nine ("Nb lesions/Brain (> = 9)") detected in the baseline MRI emerged as the most influential variable.

## 5. Discussion

The objective of this study is not to make the best predictor of the trajectory of MS, but rather to explore the amount of information provided by the baseline MRI for predicting the evolution of MS. We propose a method for creating trajectory descriptors *β*_1_ and *β*_2_ (section 3) which help us understand how MS patients’ EDSS scores change over time. *β*_1_ simplifies the description, by connecting the first and last EDSS assessments with a straight line (see Fig 4D), but it may lose important information and does not consider variations between measurements. In contrast, *β*_2_ considers these variations over time and the changes in EDSS scores between consecutive measurements. Both methods are weighted by the time between measurements. The hyperoptimized XGBoost model showed the lowest MAE, suggesting it is better at predicting patient trajectories. For *β*_1_, the MAE is 8.62 percent relative to the standard deviation, while for *β*_2_, it is 40.60 percent relative to the standard deviation. This difference can be attributed to the fact that *β*_2_ considers intermediate changes in EDSS values, including relapses in patients, which can lead to randomly occurring elevated atypical EDSS values, potentially making predictions for this descriptor more challenging. This method is a useful tool for quickly characterizing disease behaviour over time, but it introduces an error when converting categorical EDSS measurements into numbers, assuming all transitions between categories carry the same weight.

To address the limitations of *β*_1_ and *β*_2_, we present an alternative method for constructing the *EDSS*(*t*) trajectory descriptor. This new approach treats the variable as categorical, focusing on how patients change over time. The descriptor is based on EDSS measurements at five specific time points, with varying patient counts: 446, 400, 377, 352, and 279, respectively. As shown in Table 1, EDSS values are not evenly distributed across categories, with most samples having values below EDSS = 4. Consequently, we only considered categories with a minimum of ten occurrences at each time point. Table 4 compares the performance of the MLR against optimized XGBoost models using various metrics. Notably, the XGBoost model exhibited the most promising results, demonstrating AUC-ROC values ranging from 0.7354 for *EDSS*(0) to a peak of 0.9136 for *EDSS*(1). The predictions for *EDSS*(0) showed slightly lower performance compared to other time points. There are plausible reasons to think that was due to an imbalance in the sample distribution at the initial stage, where category "2" represented 28% of all samples, while category "1.5" accounted for only 3%. To handle this imbalanced-ness of the problem, several actions have been taken to mitigate the effects as using a schema for validation considering a 5-fold cross validation approach. As this initial imbalance in debut conditions produces suboptimal outcomes of the estimator only at this specific initial time point, the task of exploring techniques to address this intrinsic data imbalance is posed as an open future research study that potentially mitigate such issues improving the overall performance of predictive models for EDSS trajectory descriptors.

Several works have been published in recent years within the same domain, focusing on predicting patient evolution using MRI studies [28–30]. These studies aim to forecast disease progression at various time points according to the EDSS scale. While the prevailing literature reports AUC values ranging from 0.71 to 0.89, our findings span from 0.74 to 0.91, contingent upon the forecasted year for disease trajectory. While the resulting metrics from these works align with ours, comparisons are somewhat heterogeneous due to differences in input variables, prediction different time points, and considerations of the EDSS scale. Moreover, our proposed methodology focuses on forecasting disease progression solely utilizing derived features extracted from baseline MRI scans.

It is interesting to remark that while baseline MRI studies are a good prognostic predictor for MS, as demonstrated by the research community [31,32], the performance disparity between the classifier models EDSS(0) predicting the initial EDSS level and the EDSS(1) at one year and later, could be understood as a contribution of clinical variables such as treatment, genetics and environmental factors, to the clinical evolution and assessment of the patient.

The implementation of explainable AI methods facilitated the discovery of the core factors influencing precise decisions within the ML model. This process renders complex models understandable and accessible, even to those without advanced technical or medical knowledge. In section 4.3, SHAP was utilized to interpret the ML models, both for the regressor and the classifier. This analysis provided essential information about the internal performance of each developed predictor and descriptor, including the classification of feature importance and insights into how the values of each feature impact predictions. For the trajectory descriptors *β*1 and *β*2, significant influence was highlighted, particularly related to the age at disease diagnosis (age at onset), as observed in Fig 7. This observation aligns with findings from previous studies [33–35]. In the analysis of EDSS(t) using the XGBoost-based classifier, Fig 8 illustrates how the influence of each variable changes depending on the class being analysed for each measured time point. It is noteworthy that, in this predictor, the age of onset does not exert as much influence as in the previously analysed predictors. Instead, the most influential variable is the number of lesions greater than nine ("Nb lesions/Brain (> = 9)") detected in the baseline MRI. We found that the variable with the most significant impact in the classification models is the number of lesions greater than nine ("Nb lesions/Brain (> = 9)") in the baseline MRI. While previous works have not specifically analysed the number of lesions to predict EDSS, there are studies that have examined the prediction of EDSS at 10 years based on brain lesion volume [36], as well as others that have investigated the spatial distribution of lesions [37]. Therefore, brain lesions emerge as a crucial parameter to consider in predicting the progression of MS. The incorporation of the SHAP tool represents a significant advancement towards transparency and understanding in the context of AI and predictive modelling. This allows healthcare professionals to comprehend how the model generates prediction and make informed decisions.

In the next coming years AI will have a great impact on the clinic when it comes to making clinical decisions, prevention / diagnosis / prognosis, therapeutic efficacy, etc. This work makes an intensive use of AI algorithms for producing prognosis and decisions tools, intentionally derived exclusively from the baseline MRI, to measure the amount of information for prediction patient evolution at the debut. Enhancing the model’s effectiveness could be accomplished by incorporating longitudinal MRI data, enabling more precise and resilient predictions at each temporal instance. This approach could aid in identifying patterns in MRI images associated with disease progression. Including supplementary clinical information, such as laboratory results, genetic data, or other relevant biomarkers for MS could also enrich the models and enhance their predictive capacity. However, it is important to consider the challenges associated with collecting and integrating additional clinical data. Concerning data limitations on this study, an additional issue for future works should be addressed in terms of two main aspects, the public accessibility of the dataset employed and the inclusion of external databases to further validate the proposed method in a broader cohort. Actions on those two lines have been started but as this falls out of the scope of this paper, result will be included in future works.

## 6. Conclusions

This paper presents a new method for describing MS trajectories based on two new numerical scores (β1 and β2) and a categorical descriptor for time evolution, EDSS(t). The state-of-the-art XGBoost method was employed to predict these new trajectory descriptors using information provided in the baseline MRI study, and the results were compared with the classical models. Hyperparameter optimized XGBoost models improves the predictions of trajectories of MS patients, in both categories regressors and classifiers. In terms of best AUC-ROC, Sensitivity, Accuracy and Precision. The layer of Explainable ML facilitates understanding the reasons and variable importance within the AI models, being "Age at onset" the most significant variable in predicting trajectories *β*_1_ and *β*_2_, while "Number of lesions in the brain (> = 9)" holds the highest importance in predicting the trajectory and EDSS(t). This paper shows how AI is superior to traditional logistic regression in predicting the patient’s disability status at 1, 2, 5 and 10 years based on human-imputed data regarding specific standardized findings in a baseline MRI that could impact decisions. It is possible that the incorporation of AI analysis of MRI could further improve the model’s prediction abilities.

## Supporting information

S1 Table Characteristics of the cohort. (DOCX)

S2 Table Classifier performance on train data. (DOCX)
