# Supplementary material for: Explainable machine learning on baseline MRI predicts multiple sclerosis trajectory descriptors
Source: PLoS One. 2024 Jul 16;19(7):e0306999. doi: 10.1371/journal.pone.0306999 (PMC11251627; doi:10.1371/journal.pone.0306999)
Supplement: S1 Table — (DOCX) [file pone.0306999.s001.docx]

Supplementary Table. 1 Characteristics of the cohort employed on this work.

| **Variable** | **Value** |
| --- | --- |
| **Age at MS onset** | 31.26 ± 10.51  (mean ± standard deviation) |
| **Gender** | Male: 150  Female: 296 |
| **Number of Brain Lesions** | More than 9: 273  Less than 9: 173 |
| **Brain/Supratentorial** | 2:    359  1:     45  0:     42 |
| **Brain/Infratentorial** | 2:    174  1:    193  0:     79 |
| **Spinal cord/Cervical** | 2:    104  1:     48  0:    294 |
| **Spinal cord/Thoraco** | 2:     60  1:     39  0:    347 |
| **Optic nerve** | 2:      7  1:      5  0:    434 |
| **Gado** | 2:    105  1:    128  0:    213 |
| **Brain/Supratentorial (T1)** | 2:     94  1:     42  0:    310 |
| **Brain/Supratentorial (T1 Gado)** | 2:     69  1:    132  0:    245 |
| **Brain/Supratentorial (T2 PD)** | 2:    340  1:     35  0:     71 |
| **Brain/Infratentorial (T1)** | 2:     20  1:    108  0:    318 |
| **Brain/Infratentorial (T1 Gado)** | 2:     16  1:    176  0:    254 |
| **Brain/Infratentorial (T2 PD)** | 2:    170  1:    159  0:    117 |
| **Spinal cord/Cervical (T1)** | 2:      4  1:     29  0:    413 |
| **Spinal cord/Cervical (T1 Gado)** | 2:     25  1:      44  0:    377 |
| **Spinal cord/Cervical (T2 PD)** | 2:     98  1:     42  0:    306 |
| **Spinal cord/Thoraco (T1)** | 1:     18  0:    428 |
| **Spinal cord/Thoraco (T1 Gado)** | 2:     11  1:     28  0:    407 |
| **Spinal cord/Thoraco (T2 PD)** | 2:     56  1:     29  0:    361 |
| **Optic nerve (T2 PD)** | 2:      5  1:      4  0:    437 |
| **Nb lesions/Brain (PV)** | 3:    288  2:     10  1:     19  0:    129 |
| **FLAIR done** | 1:     35  0:    411 |
| **Nb lesions/Brain (JC)** | 1:    243  0:    203 |
| **Nb lesions/Brain (<9)** | 8:      5  7:      5  6:      4  5:      8  4:     10  3:     11  2:      7  1:     24  0:    372 |
| **Confluent** | 1:     12  0:    434 |
| **Nb lesions/Spinal** | 2:     55  1:     62  0:    329 |

Brain/Supratentorial, whether any supratentorial lesions are present; Brain/Infratentorial, whether any infratentorial lesions are present; Spinal cord/Cervical, whether any lesions are present in the cervical spinal cord; Spinal cord/Thoraco, whether any lesions are present in the thoracic spinal cord; Optic nerve, whether any lesions are present in one or both optic nerves; Gado, whether there is any gadolinium enhancement; Brain/Supratentorial (T1), whether any T1 hypointense supratentorial lesions are present; Brain/Supratentorial (T1 Gado), whether any gadolinium enhancing supratentorial lesions are present; Brain/Supratentorial (T2 PD), whether any T2 hyperintense supratentorial lesions are present; Brain/Infratentorial (T1), whether any T1 hypointense infratentorial lesions are present; Brain/Infratentorial (T1 Gado), whether any gadolinium enhancing infratentorial lesions are present; Brain/Infratentorial (T2 PD), whether any T2 hyperintense infratentorial lesions are present; Spinal cord/Cervical (T1), whether any T1 hypointense cervical lesions are present; Spinal cord/Cervical (T1 Gado), whether any gadolinium enhancing cervical lesions are present; Spinal cord/Cervical (T2 PD), whether any T2 hyperintense cervical lesions are present; Spinal cord/Thoraco (T1), whether any T1 hypointense thoracic lesions are present; Spinal cord/Thoraco (T1 Gado), whether any gadolinium enhancing thoracic lesions are present; Spinal cord/Thoraco (T2 PD), whether any T2 hyperintense thoracic lesions are present; Optic nerve (T2 PD), whether any T2 hyperintense optic nerve lesions are present; Nb lesions/Brain (PV), Number of periventricular T2 hyperintense lesions (0: none, 1: 1-9, 2: 10-29, 3: ≥30); FLAIR done, whether FLAIR sequence has been performed; Nb lesions/Brain (JC), whether there are any juxtacortical brain lesions (0: none, 1: one or more); Nb lesions/Brain (<9), Number of patients with each number of brain lesions, if lower than 9; Confluent, whether there are any T2 hyperintense confluent brain lesions; Nb lesions/Spinal, Number of patients with each number of spinal lesions 0: no spinal cord lesions, 1: one spinal cord lesion, 2: two or more spinal cord lesions.
