# Supplementary material for: Explainable machine learning on baseline MRI predicts multiple sclerosis trajectory descriptors
Source: PLoS One. 2024 Jul 16;19(7):e0306999. doi: 10.1371/journal.pone.0306999 (PMC11251627; doi:10.1371/journal.pone.0306999)
Supplement: S2 Table — (DOCX) [file pone.0306999.s002.docx]

Supplementary Table. 2 Performance Comparison of Classifier Models for Predicting

EDSS(t) on Training Dataset.

| $\boldsymbol{EDSS(t)}$ | **Models** | **Macro-AUC** | **Sensitivity** | **Accuracy** | **Precision** |
| --- | --- | --- | --- | --- | --- |
| $\boldsymbol{EDSS(0)}$ | **MLR**  **XGBoost** | 0.6941  **0.7500** | 0.2013  0.7792 | 0.3116  0.4395 | 0.2406  0.5074 |
| $\boldsymbol{EDSS(1)}$ | **MLR**  **XGBoost** | 0.6072  **0.9290** | 0.2129  0.8832 | 0.2286  0.8650 | 0.2186  0.8120 |
| $\boldsymbol{EDSS(2)}$ | **MLR**  **XGBoost** | 0.7910  **0.8812** | 0.3227  0.8934 | 0.4653  0.7721 | 0.3300  0.7980 |
| $\boldsymbol{EDSS(3)}$ | **MLR**  **XGBoost** | 0.7516  **0.9010** | 0.2397  0.7980 | 0.4058  0.8133 | 0.2170  0.8211 |
| $\boldsymbol{EDSS(4)}$ | **MLR**  **XGBoost** | 0.7502  **0.8915** | 0.2512  0.8833 | 0.4772  0.7288 | 0.2405  0.7308 |
